# Effects of exercise with or without *β*-hydroxy-*β*-methylbutyrate supplementation on muscle mass, muscle strength, and physical performance in patients with sarcopenia: a systematic review and meta-analysis

**DOI:** 10.3389/fnut.2024.1460133

**Published:** 2024-09-18

**Authors:** Yiwei Feng, Peng Chen, Tao Li, Ping Wan, Rengfei Shi

**Affiliations:** ^1^School of Exercise and Health, Shanghai University of Sport, Shanghai, China; ^2^School of Sports and Health, Shanghai Lixin University of Accounting and Finance, Shanghai, China

**Keywords:** exercise, *β*-hydroxy-β-methylbutyrate (HMB), sarcopenia, muscle strength, muscle mass, physical function, body composition

## Abstract

**Objectives:**

This systematic review and meta-analysis aimed to assess the effects of exercise with/without *β*-hydroxy-β-methylbutyrate (HMB) supplementation on muscle mass, muscle strength, physical performance, and body composition in patients with sarcopenia.

**Methods:**

A literature search for randomized controlled trials (RCTs) on the effects of exercise with or without HMB supplementation on muscle mass, muscle strength, physical performance, and body composition in patients with sarcopenia was conducted using PubMed, Web of Science, EBSCO, The Cochrane Library, EMBASE, Scopus, Science Direct, China Knowledge Resource Integrated Database (CNKI), and Wan Fang database. The search was limited to studies published up to April 2024 for each database. The outcome measures included muscle mass, muscle strength, physical performance, and body composition. The Cochrane Risk of Bias Assessment Tool was used to evaluate the quality of the included literature, and RevMan 5.4 software was employed to perform a meta-analysis of the outcome indicators.

**Results:**

Five RCTs involving 257 elderly patients with sarcopenia were included in this study. Meta-analysis showed that in terms of physical performance, exercise with HMB supplementation significantly increased gait speed in sarcopenic patients compared to the exercise combined with the placebo group (SMD = 0.48, 95% CI: 0.15 to 0.82, *p* = 0.005), but exercise combined with HMB supplementation did not have significant effects on SMI (SMD = 0.06, 95% CI: −0.20 to 0.32, *p* = 0.66), grip strength (SMD = 0.23, 95% CI: −0.05 to 0.52, *p* = 0.11), five-time chair stand test (SMD = –0.83, 95% CI: −1.88 to 0.21, *p* = 0.12), fat-free mass (SMD = 0.04, 95% CI: –0.26 to 0.35, *p* = 0.78), BMI (SMD = –0.09, 95% CI: –0.43 to 0.25, *p* = 0.60), and fat mass (SMD = 0.01, 95% CI: –0.25 to 0.27, *p* = 0.94).

**Conclusion:**

The current evidence indicates that exercise with HMB supplementation may enhance physical performance in patients with sarcopenia compared to exercise with the placebo group. However, the effects on muscle mass, muscle strength, and body composition are likely minimal. The above findings are limited by the number of included studies and require further validation through high-quality studies.

**Systematic Review Registration:**

Prospero (CRD42024500135).

## 1 Introduction

Sarcopenia is a progressive, generalized skeletal muscle disorder that is characterized by accelerated loss of muscle mass and/or muscle function (1, 2). It is recognized as a significant clinical problem that affects older adults, with the potential to negatively impact their health. Sarcopenia is associated with an increased risk of adverse outcomes, including falls, functional decline, frailty, reduced quality of life, and death in older adults (3, 4). Reduced muscle mass is associated with loss of muscle strength and decreased physical performance (5–7). It is a clinical priority to improve muscle mass, muscle strength, and physical performance in patients with sarcopenia.

There are several strategies for the prevention and treatment of sarcopenia, including exercise training, nutritional supplements, and hormone therapy (1). Exercise and nutritional supplementation interventions have been demonstrated to be effective and safe ways to improve sarcopenia (8–10). *β*-hydroxy-β-methylbutyrate (HMB), a metabolite of the branched-chain amino acid leucine, has been investigated as a potential supplement to improve muscle mass (11). Several studies have indicated that HMB can counteract sarcopenia by activating skeletal muscle protein synthesis signaling pathways and inhibiting muscle proteolysis (12, 13). However, the results of studies on the efficacy of HMB supplementation in clinical trials have been inconsistent. Some systematic reviews and meta-analyses have been conducted on the effects of HMB supplementation on muscle mass, muscle strength, and physical performance in different populations, including trained athletes, untrained individuals, and diseased populations (14–18). These studies have yielded conflicting results, with the effects of HMB supplementation on muscle mass, muscle strength, and physical performance varying across populations. Furthermore, not all studies have found a beneficial effect of HMB supplementation.

In recent years, the therapeutic effects of exercise combined with HMB supplementation in older adults with sarcopenia have been investigated. However, it has not been established whether exercise combined with HMB supplementation further improves sarcopenia compared to the exercise combined with the placebo group. For example, Meza-Valderrama et al. (19) demonstrated that supplementation with 3 g/day of Ca-HMB combined with progressive resistance exercise significantly enhanced muscle strength and physical performance in older women with sarcopenia. However, Osuka et al. (20) found that HMB supplementation did not enhance the effects of exercise on muscle mass, and muscle strength in older women with sarcopenia and that combining HMB supplementation with exercise was not a more effective strategy. Therefore, the objective of this systematic review and meta-analysis was to explore the effects of exercise with/without HMB supplementation on muscle mass, muscle strength, and physical performance in patients with sarcopenia.

## 2 Materials and methods

This study was conducted following the 2020 Preferred Reporting Items for Systematic Reviews and Meta-Analyses (PRISMA) guidelines (21) (Supplementary Table S1) and the Cochrane Handbook for Systematic Reviews of Interventions (22). Furthermore, the systematic review was registered with the International Prospective Registry of Systematic Reviews (PROSPERO): CRD42024500135. The primary processes of literature search and selection, data organization, data extraction, risk of bias assessment, statistical analysis, and other significant processes were conducted independently by two investigators (YWF and PC), with discrepancies resolved through discussion and negotiation. If no consensus was reached, the matter was discussed with the third researcher (RFS).

### 2.1 Retrieval strategy

A comprehensive search of the literature was conducted in PubMed, Web of Science, EBSCO, The Cochrane Library, EMBASE, Scopus, Science Direct, and the China Knowledge Resource Integrated Database (CNKI), as well as Wan Fang, from the time of library construction to April 1, 2024. The search strategy consisted of the following Medical Subject Headings (MeSH) terms and free words: The search terms included “sarcopenia,” “exercise,” “*β*-hydroxy-β-methylbutyric acid (HMB) “, and “randomized controlled trial.” The search strategies for each database can be viewed in Supplementary Table S2.

### 2.2 Inclusion and exclusion criteria

#### 2.2.1 Inclusion criteria

Following the principles of population, intervention, comparison, outcome, and study design (PICOS), the following inclusion criteria were established for this investigation:

1. Population: met the consensus criteria for the diagnosis of sarcopenia [e.g., refer to the diagnostic criteria of the Asian Working Group for Sarcopenia (AWGSOP) (23, 24) or European Working Group on Sarcopenia in Older People (EWGSOP) (25): low muscle mass, low muscle function, and/or decreased physical performance]. There were no restrictions on gender, socioeconomic status, race, or country.
2. Intervention: exercise combined with HMB supplementation [any form, including HMB-containing supplements, calcium (Ca-HMB) salt, and free acid (FA-HMB) forms].
3. Comparator: exercise alone or exercise combined with placebo supplementation.
4. Outcome: The primary outcome indicators were muscle mass (including skeletal muscle mass index [SMI]), muscle strength (including handgrip strength), and physical performance (including gait speed, five-time chair stand test). The secondary outcome indicators were body composition (including body mass index [BMI], fat-free mass, and fat mass).
5. Study design: The studies were randomized controlled trials (RCTs) published in English and Chinese.

Furthermore, the reference lists of the included articles were retrieved to identify any other articles that might meet the inclusion criteria.

#### 2.2.2 Exclusion criteria

Studies were excluded if they were (i) based on unavailable full text or lacked data, (ii) published in a conference paper, (iii) duplicates of previously published works, or (iv) based on flawed experimental designs and low-quality methodologies.

### 2.3 Literature screening and information extraction

The retrieved literature was imported into the software Endnote X9 and screened for inclusion and exclusion criteria. The following information was extracted from the included studies: authors, year of publication, subject, age, sample size, diagnostic criteria for sarcopenia, HMB supplementation program, type of exercise, duration of exercise, and outcome indicators.

### 2.4 Risk of bias assessment

The risk of bias in each study was assessed using the Cochrane Risk of Bias Assessment Tool (22). This tool evaluates the risk of bias in six main areas: random sequence generation, allocation concealment, blinding of investigators and subjects, blinding of study outcomes, completeness of outcome data, selective reporting of findings, and other sources of bias, for a total of seven entries. The risk of bias was evaluated. For each item, the risk of bias was determined as “low risk of bias,” “high risk of bias,” or “unclear risk of bias” according to the guidelines for risk of bias assessment.

### 2.5 Statistical analysis

A meta-analysis was conducted using Revman 5.4 software (Version 5.4, Copenhagen: The Nordic Cochrane Center, The Cochrane Collaboration, 2020). As the outcome indicators in the literature included in this study were continuous variables and were measured differently, the standardized mean difference (SMD) was used as the effect size indicator, with 95% confidence intervals (CI) calculated. Inter-study heterogeneity was analyzed using the *χ*^2^ test and *I*^2^ values. If the *p*-value is greater than 0.05 and the *I*^2^ value is less than 50%, it can be concluded that the heterogeneity between the results of each study is minimal and that the fixed-effects model can be employed for analysis. Conversely, if the *p*-value is less than 0.05 and the *I*^2^ value is greater than 50%, it can be inferred that the heterogeneity between the results of each study is significant and that the random-effects model is required for analysis. To ascertain the influence of individual studies on the overall pooled results, a sensitivity analysis was conducted using the method of excluding individual papers one by one. A statistically significant difference between the two groups was indicated by *p* < 0.05. The stability of this study was evaluated by sensitivity analysis. Funnel plots were produced for outcome indicators with ≥10 included literature to analyze the presence of publication bias.

## 3 Results

### 3.1 Literature search results

**Figure 1:**
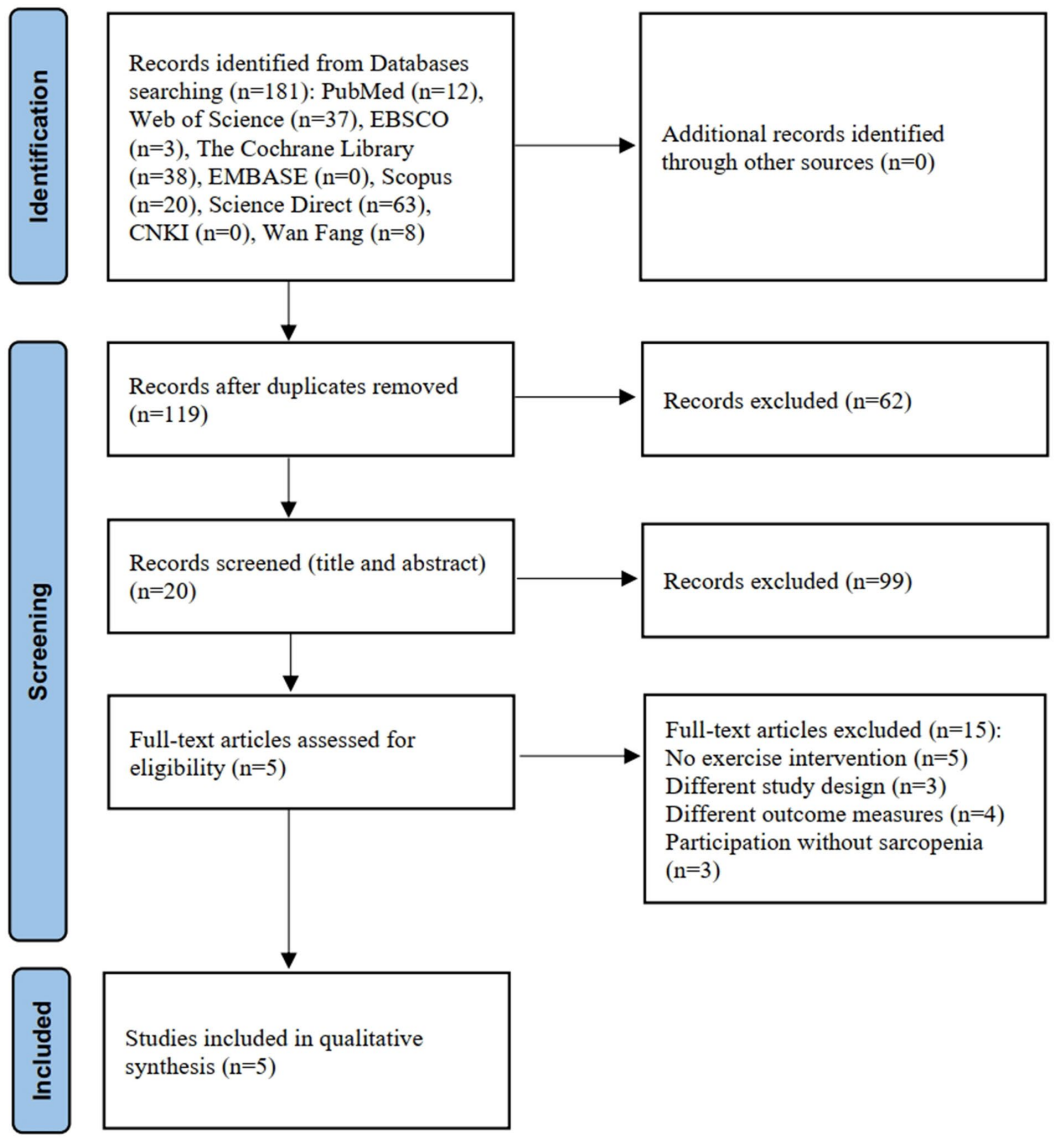
Flow chart of the search process and study selection.

A total of 181 articles were identified through the literature search. After removing duplicates and screening titles and abstracts, 161 articles were excluded. A further 15 articles were removed after assessment of the full text. Finally, after reading the full text, five studies (19, 20, 26–28) were included for quantitative analysis. The detailed literature screening process is depicted in Figure 1, and examples of the excluded literature can be viewed in Supplementary Table S3.

### 3.2 Basic characteristics of the literature included

A total of five studies were included in this systematic review and meta-analysis, from China (26–28), Japan (20), and Spain (19), with one document in Chinese (27) and the rest in English (19, 20, 26, 28). A total of 257 subjects who were older adults with a definite diagnosis of sarcopenia were enrolled in the studies, with 130 subjects in the exercise and HMB combination group and 127 subjects in the control group. In three of the five trials (19, 20, 28), HMB supplementation was reported to be administered in the form of calcium salts (Ca-HMB), while the other two trials (26, 27) did not mention the specific form of HMB supplementation. Except for one study (20), in which the dose of HMB supplementation was 1.5 g/day, the remaining studies (19, 26–28) administered 3 g/day. All studies employed resistance training (RT) as the exercise regimen, with the intervention period lasting approximately 12 weeks. The intervention period was essentially focused on 12 weeks, with an average of 40–60 min of exercise 2–3 times a week. Four studies (20, 26–28) used the diagnostic criteria published by AWGSOP, while one study (19) used the diagnostic criteria published by EWGSOP. A detailed overview of the study characteristics and data is presented in Table 1.

**Table 1:** Basic information of the literature included in the meta-analysis.

Year	Authors/references	Country	Diagnostic criteria	Participants	Group: years, mean ± standard deviation	Sample size	Intervention group supplement	Control group supplement	Type of exercise	Duration of exercise intervention	Outcome measures
2021	Osuka et al. (20)	Japan	AWGSOP 2014 (23)	Women with sarcopenia	E + H: 73.5 ± 4.2; E + P: 71.8 ± 4.1	E + H: *n* = 36; E + P: *n* = 38	Ca-HMB (1.5 g/day)	Placebo	RT	50 min each time,12-week, twice weekly	SMI, FFM, BMI, FM, HS, FTCST, GS
2022	Han et al. (26)	China	AWGSOP 2014 (23)	Elderly patients with sarcopenia after hip replacement	E + H: 78.3 ± 5.9; E + P: 77.1 ± 6.5	E + H: *n* = 43; E + P: *n* = 45	HMB (3 g/day)	Without HMB supplement	Progressive RT	Once every 2–3 days, 3 months	SMI, FM, HS
2023	Yang et al. (28)	China	AWGSOP 2019 (24)	Older adults aged ≥60 years with sarcopenia	E + H: 72.89 ± 7.02; E + P: 71.44 ± 5.22	E + H: *n* = 18; E + P: *n* = 16	Ca-HMB (3 g/day)	Placebo	RT	40 min each time,12-week, twice a week	SMI, FFM, HS, FTCST, GS
2023	Meza-Valderrama et al. (19)	Spain	EWGSOP 2019 (25)	Older adults with sarcopenia after discharge	E + H: 81.8 ± 8.8; E + P: 81.3 ± 10.2	E + H: *n* = 17; E + P: *n* = 15	Ca-HMB (3 g/day)	Placebo	Progressive RT	One hour each time,12-week, three times per week	FFM, BMI, FM, HS, GS
2022	Chen et al. (27)	China	AWGSOP 2019 (24)	Elderly patients with sarcopenia	E + H: 74.31 ± 6.03; E + P: 72.23 ± 5.07	E + H: *n* = 16; E + P: *n* = 13	HMB (3 g/day)	Placebo	RT	40 min each time, twice a week, 12-week	SMI, FFM, BMI, FM

M, male; F, female; E, exercise; H, HMB; P, placebo; RT, resistance training; AWGSOP, Asian Working Group for Sarcopenia; EWGSOP, European Working Group on Sarcopenia in Older People; SMI, skeletal muscle index; FFM, fat-free mass; BMI, body mass index; FM, fat mass; HS, handgrip strength; FTCST, five-time chair stand test; GS, gait speed.

### 3.3 Risk of bias assessment

**Figure 2:**
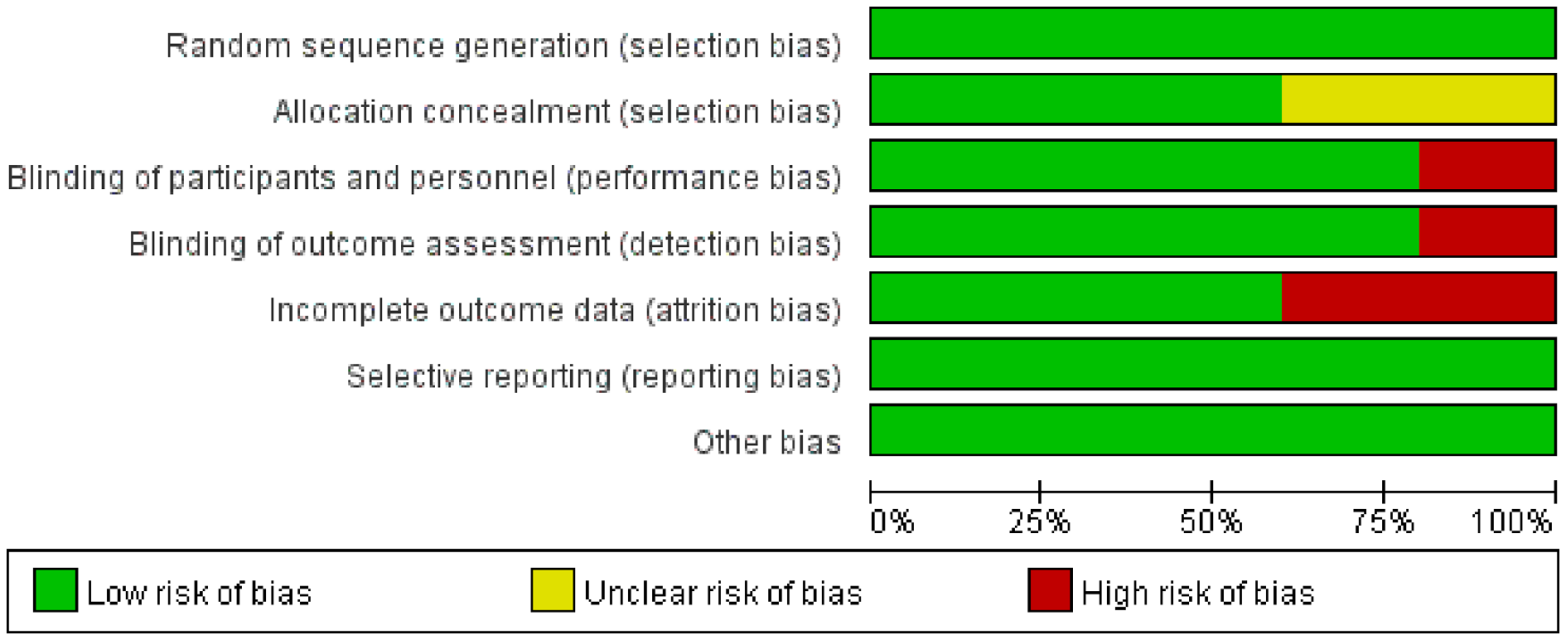
Risk of bias graph. Green color indicates a low risk of bias, yellow color indicates an unclear risk of bias, and red color indicates a high risk of bias.

**Figure 3:**
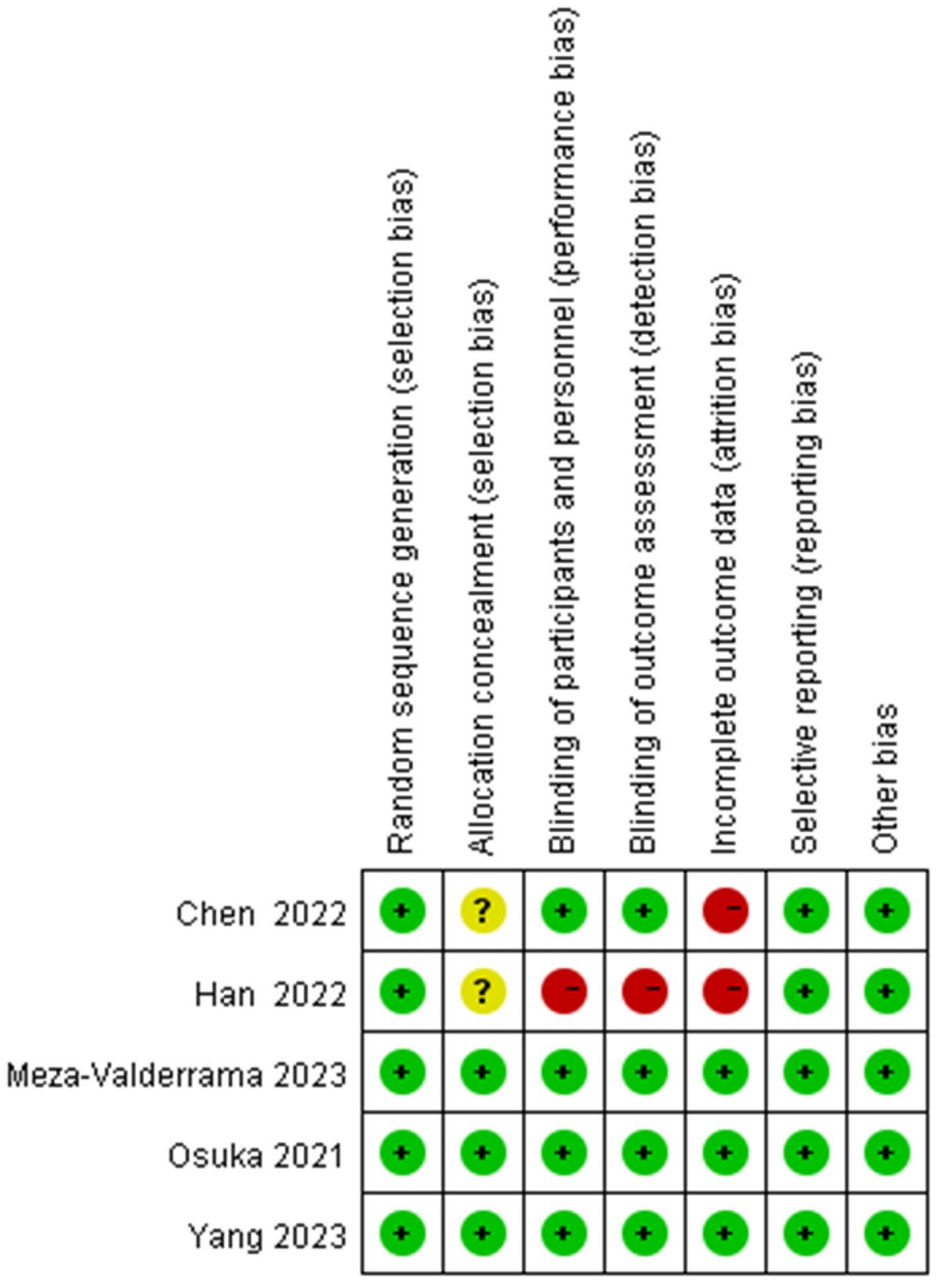
Risk of bias summary. “–” For high risk of bias, “?” for unclear risk of bias, and “+” for low risk of bias.

Figures 2, 3 show the risk of bias for the included studies. In terms of random sequence generation, five studies (19, 20, 26–28) reported specific random sequence generation methods, mainly the random number table method and the computerized random sequence method. In terms of allocation concealment, three studies (19, 20, 28) used allocation concealment. And in terms of blinded assessment, four studies (19, 20, 27, 28) used a double-blind design and one study (26) did not mention its blinding design. In terms of completeness of outcome data, five studies (19, 20, 26–28) had case shedding, three studies (19, 20, 28) used an intention-to-treat (ITT) analysis and were judged to be at low risk, while the other two studies (26, 27) did not use a rational approach to missing data and were judged to be at high risk of bias. And in terms of selective reporting of results and other sources of bias, five studies (19, 20, 26–28) were free from selective reporting and conflict of interest.

### 3.4 The results of the meta-analysis

#### 3.4.1 Skeletal muscle index

**Figure 4:**
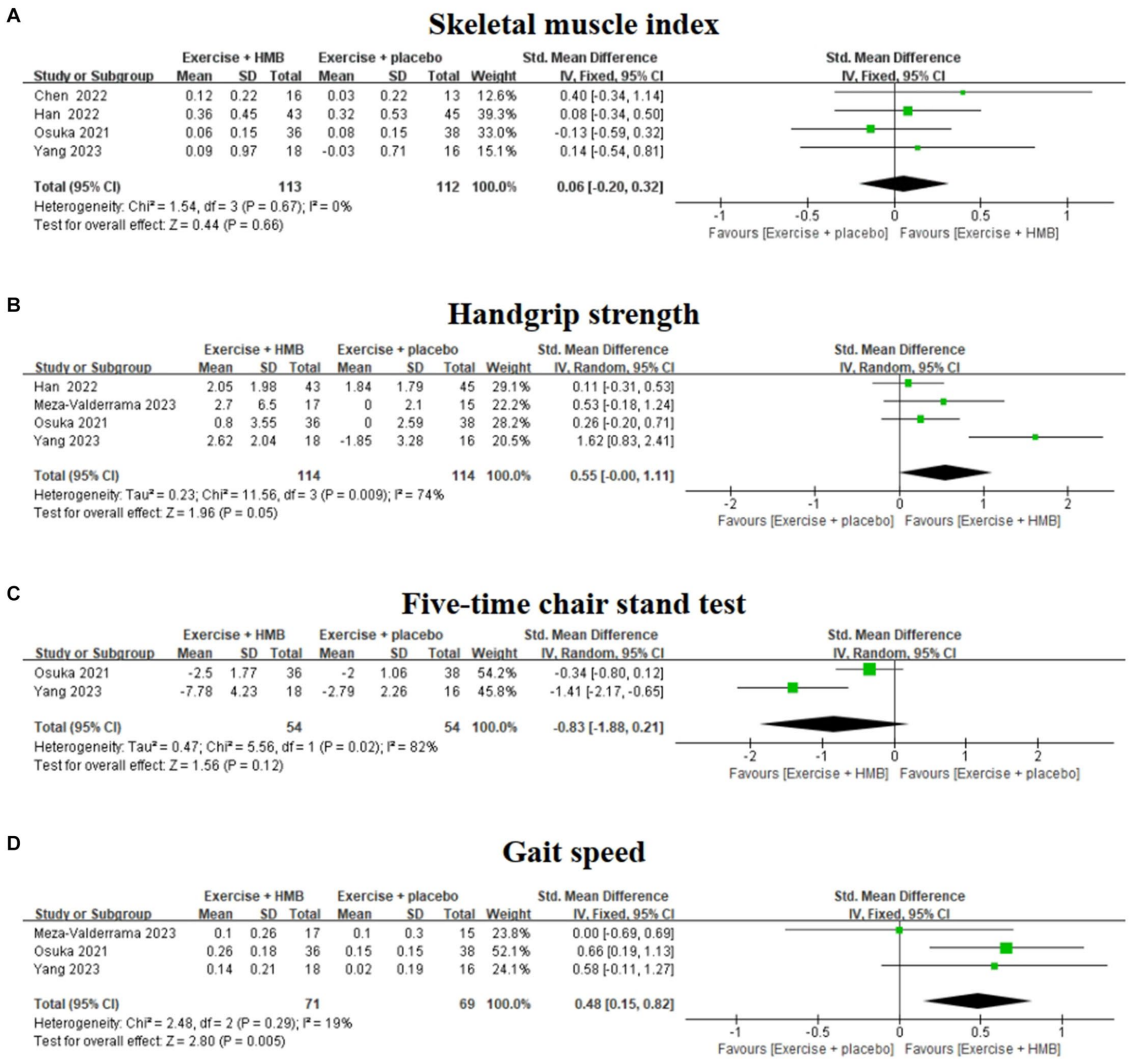
Forest plot of the effect of exercise with/without HMB supplementation on SMI **(A)**, handgrip strength **(B)**, five-time chair stand test **(C)**, and gait speed **(D)** in patients with sarcopenia.

SMI is the ratio of skeletal muscle mass to height squared, which allows objective quantitative comparison of differences in muscle mass between individuals and is an important index for assessing muscle mass and diagnosing sarcopenia (29, 30). Among the included studies, four studies (20, 26–28) evaluated the effect of exercise with/without HMB supplementation on SMI in sarcopenia (Figure 4A). The homogeneity among the four studies was good (*I*^2^ = 0%, *p* = 0.67), so meta-analysis was performed using a fixed effects model. The results showed no significant difference in SMI between the exercise combined with the HMB supplementation group and the exercise combined with the placebo group (SMD = 0.06, 95% CI: −0.20 to 0.32, *p* = 0.66).

#### 3.4.2 Handgrip strength

Handgrip strength is an indicator to assess upper limb muscle strength in patients with sarcopenia (30). Four studies (19, 20, 26, 28) evaluated the effect of exercise with/without HMB supplementation on grip strength in patients with sarcopenia (Figure 4B). The heterogeneity among the four studies was high (*I*^2^ = 74%, *p* = 0.009), so a meta-analysis was performed using a random effects model. The results showed that there was no significant difference in handgrip strength between the exercise combined with the HMB supplementation group and the exercise combined with the placebo group (SMD = 0.55, 95% CI: −0.00 to 1.11, *p* = 0.05).

Sensitivity analysis identified the study by Yang et al. (28) as the main source of high heterogeneity, and after removing this study, a meta-analysis of the remaining data showed a significant reduction in between-study heterogeneity (*I*^2^ = 0%, *p* = 0.60), but no significant change in the overall effect size (SMD = 0.23, 95% CI: −0.05 to 0.52, *p* = 0.11). It is suggested that exercise combined with HMB does not significantly improve grip strength in patients with sarcopenia.

#### 3.4.3 Five-time chair stand test

The five-time chair stand test is a commonly used method to reflect physical performance and muscle strength in patients with sarcopenia (30). Two studies (20, 28) evaluated the effect of exercise with/without HMB supplementation on the five-time chair stand test in patients with sarcopenia (Figure 4C). The heterogeneity between the results of the two studies was high (*I*^2^ = 82%, *p* = 0.02), so a meta-analysis was performed using a random effects model. The results showed that there was no significant difference between the exercise combined with the HMB group and the exercise combined with the placebo group in the five-time chair stand test (SMD = –0.83, 95% CI: −1.88 to 0.21, *p* = 0.12).

#### 3.4.4 Gait speed

Gait speed is a commonly used indicator of physical performance in patients with sarcopenia (30). Three studies (19, 20, 28) evaluated the effect of exercise with/without HMB supplementation on gait speed in patients with sarcopenia (Figure 4D). The heterogeneity among the results of the three studies was low (*I*^2^ = 19%, *p* = 0.29), so meta-analyses were performed using a fixed-effects model. The results showed a significant difference in gait speed between the exercise combined with the HMB supplementation group and the exercise combined with the placebo group (SMD = 0.48, 95% CI: 0.15 to 0.82, *p* = 0.005).

#### 3.4.5 Fat-free mass

**Figure 5:**
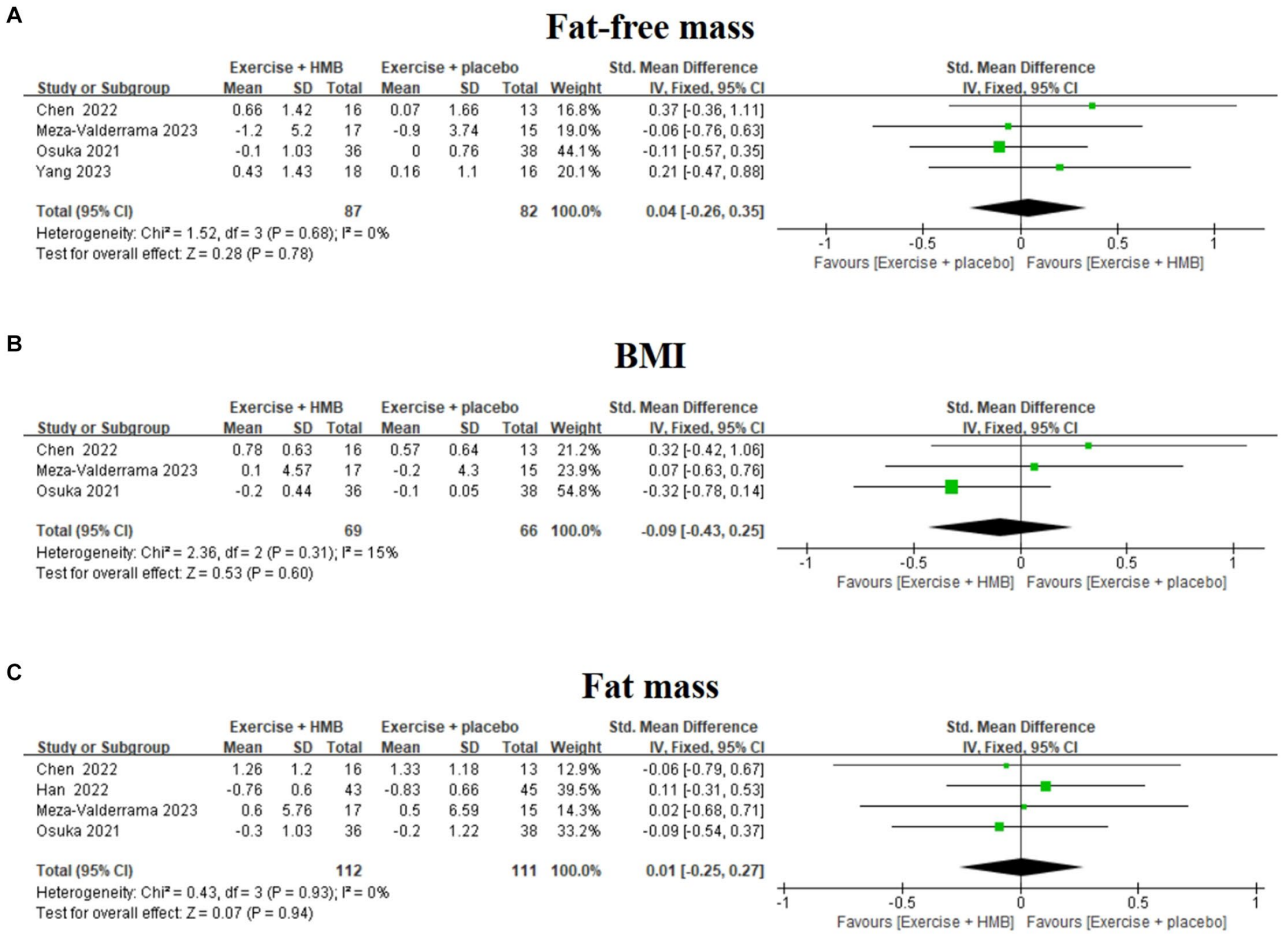
Forest plot of the effect of exercise with/without HMB supplementation on fat-free mass **(A)**, BMI **(B)**, and fat mass **(C)** in patients with sarcopenia.

Fat-free mass, BMI, and fat mass are indicators to assess body composition in patients with sarcopenia (31, 32). Four studies (19, 20, 27, 28) evaluated the effect of exercise with/without HMB supplementation on fat-free mass in patients with sarcopenia (Figure 5A). The homogeneity among the results of the four studies was good (*I*^2^ = 0%, *p* = 0.68), so a fixed-effects model was used for meta-analysis. The results showed that there was no significant difference in fat-free mass between the exercise combined with the HMB supplementation group and the exercise combined with the placebo group (SMD = 0.04, 95% CI: −0.26 to 0.35, *p* = 0.78).

#### 3.4.6 BMI

Three studies (19, 20, 27) evaluated the effect of exercise with/without HMB supplementation on BMI in patients with sarcopenia (Figure 5B). The heterogeneity among the results of the three studies was low (*I*^2^ = 15%, *p* = 0.31), so meta-analyses were performed using a fixed-effects model. The results showed that there was no significant difference in BMI between the exercise combined with the HMB supplementation group and the exercise combined with the placebo group (SMD = –0.09, 95% CI: −0.43 to 0.25, *p* = 0.60).

#### 3.4.7 Fat mass

Four studies (19, 20, 26, 27) evaluated the effect of exercise with/without HMB supplementation on adiposity in patients with sarcopenia (Figure 5C). The homogeneity between the results of the four studies was good (*I*^2^ = 0%, *p* = 0.93), and therefore meta-analyses were performed using a fixed-effects model. The results showed that there was no significant difference in fat mass between the exercise combined with the HMB supplementation group and the exercise combined with the placebo group (SMD = 0.01, 95% CI: −0.25 to 0.27, *p* = 0.94).

### 3.5 Sensitivity analysis

To further explore the source of heterogeneity in the literature, sensitivity analyses were performed on the above outcome indicators, excluding individual papers one at a time, and the overall effect size did not change significantly, suggesting that the results of the meta-analysis are relatively stable.

### 3.6 Analysis of publication bias

The use of funnel plots for publication bias analysis is usually not recommended when the meta-analysis includes fewer than 10 articles (33). This meta-analysis included a limited number of articles (five studies), so the funnel plot was not used for publication bias analysis.

## 4 Discussion

Whether the combination of exercise and HMB supplementation leads to a “1 + 1 ≥ 2” or “1 + 1 < 2” effect in clinical trials remains inconclusive. Based on the five studies included so far, we found that in terms of physical performance, exercise combined with HMB supplementation significantly increased gait speed in patients with sarcopenia. In other measures, exercise combined with HMB supplementation did not have a significant effect on SMI, handgrip strength, five-time chair-stand test, BMI, fat-free mass, and fat mass in patients with sarcopenia.

The definition and diagnostic criteria for sarcopenia have undergone several international updates and refinements (34). Several groups have proposed definitions of sarcopenia, including the EWGSOP (25), the Australian and New Zealand Society for Sarcopenia and Frailty Research (ANZSSFR) (35), the AWGSOP (24), and the Sarcopenia Definition and Outcomes Consortium (SDOC) (36). Different organizations propose different definitions, diagnostic criteria, and thresholds due to differences in ethno-demographic characteristics (37). Recently, the Global Leadership Initiative in Sarcopenia (GLIS) created the first global conceptual definition of sarcopenia: muscle mass, strength, and muscle-specific strength are considered “components of sarcopenia,” whereas impaired physical function is considered an outcome rather than a component of sarcopenia (34). In any case, muscle strength, muscle mass, and physical performance remain important indicators for the diagnosis of sarcopenia (38).

Muscle strength can be measured using handgrip strength, leg extension strength, and the five-time chair stand test. Muscle mass and body composition can be measured using bioelectrical impedance analysis (BIA), dual-energy X-ray absorptiometry (DXA), magnetic resonance imaging (MRI), and bioelectrical impedance analysis (BIA), and assessed using indicators such as SMI, skeletal muscle mass, fat-free mass, lean soft tissue (LST), and more (38–40). It is important to note that LST is the sum of water, total protein, carbohydrates, nonfat lipids, and soft tissue minerals in the body; skeletal muscle mass is a key component of LST; and fat-free mass is formed by skeletal and nonskeletal muscles, organs, connective tissues, and bones, and is the sum of LST and bone mineral components (40, 41).

Our results suggest that exercise combined with HMB supplementation does not further improve muscle mass and strength in older adults with sarcopenia compared with exercise combined with placebo supplementation. Of the five included studies, Yang et al. (28) found that HMB supplementation enhanced the effects of RT on muscle mass and muscle strength in older adults with sarcopenia. Meza-Valderrama et al. (19) found that progressive RT combined with supplementation with 3 g/day of Ca-HMB significantly improved muscle strength after the acute phase of hospitalization in older women with sarcopenia. Han et al. (26) found that RT with or without HMB supplementation significantly increased muscle mass and muscle strength after hip arthroplasty in elderly patients with femoral neck fracture and sarcopenia. Chen et al. (27) found that HMB combined with resistance exercise improved muscle mass and muscle strength in elderly patients with sarcopenia. Only the study by Osuka et al. (20) reported that exercise combined with HMB supplementation (1.5 g/day) did not improve muscle mass and muscle strength in older adults with sarcopenia. The HMB supplementation dose in the study by Osuka et al. (20) was relatively low (1.5 g/day Ca-HMB) compared with the supplementation dose (3 g/day) in four other studies (19, 26–28), and we hypothesized that the HMB supplementation dose may have contributed to the differences in supplementation effects. Due to the large sample size and high weight proportion of the study by Osuka et al. (20), the positive effects of the other four studies (19, 26–28) were offset when the effect sizes were combined with the other studies. In addition, although potential synergies between exercise and HMB supplementation remain unclear due to sample size and group limitations, the four studies (19, 26–28) still suggest resistance exercise and HMB supplementation as potential treatments for sarcopenia.

Physical performance in patients with sarcopenia can be assessed using methods such as gait speed, the Short Physical Performance Battery (SPPB), and the Time-up and Go (TUG) test (38–40). One of our main findings is that exercise combined with HMB supplementation may help improve physical performance in patients with sarcopenia compared with exercise combined with placebo supplementation. Of the four included studies, Yang et al. (28), Meza-Valderrama et al. (19), Chen et al. (27), and Osuka et al. (20) all found that HMB supplementation improved the physical performance of RT in older adults with sarcopenia. A systematic review (42) also showed that HMB supplementation improved muscle function in older adults with sarcopenia and frailty. Therefore, HMB supplementation during RT may improve physical performance in patients with sarcopenia. Moreover, additional future research is needed to fully understand the role of HMB supplementation with RT on physical performance in patients with sarcopenia.

The present meta-analysis showed that exercise combined with HMB supplementation did not result in significant changes in body composition in patients with sarcopenia compared with the exercise combined with the placebo group. However, a review showed that HMB supplementation benefits body composition in bedridden or sedentary older adults due to its anti-catabolic properties (13). Stout et al. (43) found that 24 weeks of Ca-HMB supplementation alone did not reduce fat mass in older adults and that Ca-HMB supplementation combined with RT resulted in a significant reduction in fat mass in older adults. In the Vukovich et al. study, adults aged 70 participated in an 8-week, 5-day-per-week progressive resistance training program while supplementing with HMB or placebo. The results showed an increase in fat-free mass and a decrease in percent body fat in older adults in the HMB group compared to the placebo group, confirming that HMB supplementation combined with exercise can alter body composition in older adults (44). Animal and cellular studies have suggested that Ca-HMB increases muscle fiber metabolism and fat utilization, improves fatty acid oxidation in adipocytes and muscle cells, and is expected to reduce adiposity and increase insulin sensitivity (45). Currently, sarcopenia and sarcopenic obesity are major public health challenges in the elderly. In addition, the prevalence of sarcopenia is strongly influenced by fat-free adipose tissue (FFAT). Of the five studies included, Han et al. (26) used DEX to test body composition in older adults, Yang et al. (28), Meza-Valderrama et al. (19), Chen et al. (27), and Osuka et al. (20) used BIA. Approximately 85% of adipose tissue is fat, and approximately 15% consists of FFAT. Adipose-free adipose tissue may need to be taken into account when measuring lean body mass in the extremities using DXA, and uncorrected DXA-derived FFAT may underestimate the true prevalence of sarcopenia (46, 47). Although our results did not show a benefit of exercise combined with HMB supplementation on body composition in patients with sarcopenia, the combination of HMB supplementation and resistance exercise may still be a potential strategy to improve sarcopenia and obesity based on the aforementioned studies (12).

A variety of factors can contribute to the development of sarcopenia, which can be categorized into two main types: primary sarcopenia and secondary sarcopenia. Primary sarcopenia is primarily associated with aging and has no other apparent cause. Secondary sarcopenia, on the other hand, is due to a variety of non-age-related factors, such as diseases, medications, malnutrition, and lifestyle (1, 25, 48). The five studies we included all included patients with sarcopenia. According to our speculation, the study by Osuka et al. (20), Yang et al. (28), and Chen et al. (27) may have been conducted on patients with primary sarcopenia, and the study by Han et al. (26) and Meza-Valderrama et al. (19) may have been conducted mainly on patients with secondary sarcopenia. Due to the limited amount of literature, we did not analyze subgroups of patients with different types of sarcopenia according to etiology. Patients with secondary sarcopenia may have a greater need for nutritional supplementation than patients with primary sarcopenia. Because daily dietary intake was not controlled in these studies, we speculate that nutrients in the daily diet may have reduced the effect of supplemental HMB.

Resistance exercise has been shown to increase skeletal muscle mass, muscle strength, and exercise capacity in patients with sarcopenia (49–51). Resistance exercise is an excellent and cost-effective treatment modality. The exercise regimen in all of our included studies (19, 20, 26–28) was resistance exercise, probably because HMB supplements achieve better results when combined with resistance exercise. The included studies were mainly performed with the help of tools such as elastic bands and equipment, under the supervision of a therapist, and the intervention period was focused on 12 weeks, with an average of 40–60 min of exercise 2–3 times a week. Among them, Han et al. (26) in their study performed graded training according to the model of progressive RT for healthy adults developed by the American College of Sports Medicine. The progressive RT approach is characterized by individualized, progressive intensity, moderate to high intensity (60–85% 1RM), and can be used as a recommended form of exercise for patients with sarcopenia (52). Although RT was chosen as the exercise modality in all included studies, there were differences in training groups, frequency, and intensity between studies. It is not clear how effective other different exercise modalities (e.g., endurance exercise, multimodal exercise (10)) combined with HMB supplementation are in intervening on muscle mass, muscle strength, physical performance, and body composition in patients with sarcopenia.

Another important recommendation for the treatment and/or prevention of sarcopenia is nutritional supplementation (53). Several previous studies have recognized HMB supplementation as a nutritional approach to increase skeletal muscle protein synthesis in healthy or frail older adults (12, 13, 54). *In vitro* studies have shown that HMB effectively ameliorates muscle atrophy, increases muscle production, decreases muscle cell apoptosis, and positively affects muscle protein turnover (55). Loss of muscle mass in sarcopenia patients is associated with an imbalance between muscle protein synthesis and catabolism, and HMB may increase muscle protein synthesis by activating the mammalian target of rapamycin (mTOR) pathway. In addition, HMB may increase muscle survival by inhibiting the ubiquitin-proteasome and autophagy-lysosome pathways, inhibiting skeletal muscle protein hydrolysis, and suppressing skeletal muscle cell apoptosis (12, 56).

The most commonly used dose of HMB in current studies is 3 g/day, which is considered safe and feasible (17, 56). Because only 5% of leucine is metabolized to HMB in muscle cells, additional HMB supplementation is required in older adults to ensure adequate HMB intake (12). Most of the trials included in this systematic review and meta-analysis used HMB doses of 3 g/day, and no trials reported adverse effects in subjects.

To our knowledge, this is the first meta-analysis to examine the effects of exercise with/without HMB supplementation on muscle mass, muscle strength, physical performance, and body composition in patients with sarcopenia. Our novelty lies in the fact that we investigated whether the nutritional supplement HMB has a synergistic or additive effect on exercise, i.e., whether HMB supplementation enhances the therapeutic effect of exercise in patients with sarcopenia. We compared recent studies on similar topics. For example, a systematic review and meta-analysis conducted by Courel-Ibáñez et al. (16) in 2019 examined the effects of HMB supplementation on the health of older adults in addition to exercise. Ten RCTs were included, and the study showed that supplementation with HMB in combination with exercise had no or less effect on improving body composition, muscle strength, or physical performance in adults aged 50–80 years compared with exercise combined with a placebo group, which is close to our conclusions. In 2022, Lin et al. (57) found that HMB supplementation and supplements containing HMB components helped to improve muscle strength in older adults. Overall, there are some discrepancies between the results of recently published meta-analyses and our results, which may be explained by differences in the included study populations, intervention modalities, and outcome indicators.

This review has several limitations. First, the number of articles and sample size included in this review are small, which may affect the accuracy of the meta-analysis results, and more sufficient research evidence is needed in the future. Second, the limited number of outcome indicators in the studies included in this review may affect the reliability of the results. Third, English and Chinese databases were searched for this review, but articles in other languages were excluded.

## 5 Conclusion

In summary, this systematic review and meta-analysis suggest that exercise combined with HMB supplementation may help improve physical performance in patients with sarcopenia compared with exercise combined with placebo, but the effects on muscle mass, muscle strength, and body composition may be small. Given the limitations of existing studies’ number, quality, and heterogeneity, more large-sample, multicenter, high-quality RCTs are needed for further research.

## Data Availability

The original contributions presented in the study are included in the article/Supplementary material, further inquiries can be directed to the corresponding authors.
