# Supplementary material for: Effects of exercise with or without β-hydroxy-β-methylbutyrate supplementation on muscle mass, muscle strength, and physical performance in patients with sarcopenia: a systematic review and meta-analysis
Source: Front Nutr. 2024 Sep 18;11:1460133. doi: 10.3389/fnut.2024.1460133 (PMC11444964; doi:10.3389/fnut.2024.1460133)
Supplement: Supplementary file 1 [file Data_Sheet_1.zip › Supplementary_Material/Supplementary_Material.docx]

Supplementary Material

**Effects of** **exercise with or without β-hydroxy-β-methylbutyrate (HMB)**

**supplementation on muscle mass, muscle strength, and physical**

**performance in patients with sarcopenia: a systematic review and meta-analysis**

Yiwei Feng ^1^, Peng Chen ^1^, Tao Li ^1^, Ping Wan ^2*^ and Rengfei Shi ^1*^

^1^ School of Exercise and Health, Shanghai University of Sport, Shanghai 200438, China

^2^ School of Sports and Health, Shanghai Lixin University of Accounting and Finance, Shanghai 201209, China

***Correspondence:**Corresponding Author 1:

Ping Wan

20029306@lixin.edu.cn

Corresponding Author 2:

Rengfei Shi

rfshi@sus.edu.cn

# Supplementary Tables

# Table S1. PRISMA 2020 Checklist

Please see the separate Word document named " PRISMA 2020 Checklist ".

# Table S2. Example Search Strategy

| **Database** | **Search strategy** | **Results** |
| --- | --- | --- |
| **Pubmed** | ("Sarcopenias"[Title/Abstract] OR "Sarcopenia"[MeSH Terms]) AND (("Exercise"[MeSH Terms] OR ("Exercises"[Title/Abstract] OR "physical activity"[Title/Abstract] OR "activities physical"[Title/Abstract] OR "activity physical"[Title/Abstract] OR "physical activities"[Title/Abstract] OR "exercise physical"[Title/Abstract] OR "exercises physical"[Title/Abstract] OR "physical exercise"[Title/Abstract] OR "physical exercises"[Title/Abstract] OR "acute exercise"[Title/Abstract] OR "acute exercises"[Title/Abstract] OR "exercise acute"[Title/Abstract] OR "exercises acute"[Title/Abstract] OR "exercise isometric"[Title/Abstract] OR "exercises isometric"[Title/Abstract] OR "isometric exercises"[Title/Abstract] OR "isometric exercise"[Title/Abstract] OR "exercise aerobic"[Title/Abstract] OR "aerobic exercise"[Title/Abstract] OR "aerobic exercises"[Title/Abstract] OR "exercises aerobic"[Title/Abstract] OR "exercise training"[Title/Abstract] OR "exercise trainings"[Title/Abstract] OR "training exercise"[Title/Abstract] OR (("education"[MeSH Subheading] OR "education"[All Fields] OR "Training"[All Fields] OR "education"[MeSH Terms] OR "train"[All Fields] OR "train s"[All Fields] OR "trained"[All Fields] OR "training s"[All Fields] OR "Trainings"[All Fields] OR "trains"[All Fields]) AND "Exercise"[Title/Abstract]))) AND ("beta-hydroxy-beta-methylbutyrate"[Title/Abstract] OR "beta-hydroxy-beta-methylbutyrate"[Title/Abstract] OR "beta-hydroxy-beta-methylbutyrate"[Title/Abstract] OR "3 hydroxyisovaleric acid"[Title/Abstract] OR "HMB"[Title/Abstract] OR "beta-hydroxyisovaleric acid"[Title/Abstract] OR "calcium beta hydroxy beta methylbutyrate"[Title/Abstract] OR "Ca-HMB"[Title/Abstract] OR "beta-hydroxyisovaleric acid"[Supplementary Concept])) AND ("randomized controlled trial"[Publication Type] OR "randomized"[Title/Abstract] OR "placebo"[Title/Abstract]) | 12 |
| **EMBASE** | ("Sarcopenias" OR "Sarcopenia") AND ("Exercise" OR "Exercises" OR "physical activity" OR "activities physical" OR "activity physical" OR "physical activities" OR "exercise physical" OR "exercises physical" OR "physical exercise" OR "physical exercises" OR "acute exercise" OR "acute exercises" OR "exercise acute" OR "exercises acute" OR "exercise isometric" OR "exercises isometric" OR "isometric exercises" OR "isometric exercise" OR "exercise aerobic" OR "aerobic exercise" OR "aerobic exercises" OR "exercises aerobic" OR "exercise training" OR "exercise trainings" OR "training exercise" OR "Training" OR "train" OR "trained" OR "Trainings" OR "trains") AND ("beta-hydroxy-beta-methylbutyrate" OR "beta-hydroxy-beta-methylbutyrate" OR "beta-hydroxy-beta-methylbutyrate" OR "3 hydroxyisovaleric acid" OR "HMB" OR "beta-hydroxyisovaleric acid" OR "calcium beta hydroxy beta methylbutyrate" OR "Ca-HMB" OR "beta-hydroxyisovaleric acid") AND ("randomized controlled trial" OR "randomized" OR "placebo") | 0 |
| **Scopus** | ("Sarcopenias" OR "Sarcopenia") AND ("Exercise" OR "Exercises" OR "physical activity" OR "activities physical" OR "activity physical" OR "physical activities" OR "exercise physical" OR "exercises physical" OR "physical exercise" OR "physical exercises" OR "acute exercise" OR "acute exercises" OR "exercise acute" OR "exercises acute" OR "exercise isometric" OR "exercises isometric" OR "isometric exercises" OR "isometric exercise" OR "exercise aerobic" OR "aerobic exercise" OR "aerobic exercises" OR "exercises aerobic" OR "exercise training" OR "exercise trainings" OR "training exercise" OR "Training" OR "train" OR "trained" OR "Trainings" OR "trains") AND ("beta-hydroxy-beta-methylbutyrate" OR "beta-hydroxy-beta-methylbutyrate" OR "beta-hydroxy-beta-methylbutyrate" OR "3 hydroxyisovaleric acid" OR "HMB" OR "beta-hydroxyisovaleric acid" OR "calcium beta hydroxy beta methylbutyrate" OR "Ca-HMB" OR "beta-hydroxyisovaleric acid") AND ("randomized controlled trial" OR "randomized" OR "placebo") | 20 |
| **Web of science** | #1: Exercises (Topic) OR Physical Activity (Topic) OR Activities, Physical (Topic) OR Activity, Physical (Topic) OR Physical Activities (Topic) OR Exercise, Physical (Topic) OR Exercises, Physical (Topic) OR Physical Exercise (Topic) OR Physical Exercises (Topic) OR Acute Exercise (Topic) OR Acute Exercises (Topic) OR Exercise, Acute (Topic) OR Exercises, Acute (Topic) OR Exercise, Isometric (Topic) OR Exercises, Isometric (Topic) OR Isometric Exercises (Topic) OR Isometric Exercise (Topic) OR Exercise, Aerobic (Topic) OR Aerobic Exercise (Topic) OR Aerobic Exercises (Topic) OR Exercises, Aerobic (Topic) OR Exercise Training (Topic) OR Exercise Trainings (Topic) OR Training, Exercise (Topic) OR Trainings, Exercise (Topic) OR exercise (Topic) and Preprint Citation Index (Exclude – Database)  #2: sarcopenia (Topic) OR Sarcopenias (Topic) and Preprint Citation Index (Exclude – Database)  #3: Beta-hydroxy-beta-methylbutyrate (Topic) OR beta-hydroxy-beta-methylbutyrate (Topic) OR beta-hydroxy beta-methylbutyrate (Topic) OR HMB-d6 (Topic) OR beta hydroxy beta methylbutyrate (Topic) OR 3-hydroxyisovaleric acid (Topic) OR HMB (Topic) OR beta-hydroxyisovaleric acid (Topic) OR calcium beta-hydroxy-beta-methylbutyrate (Topic) OR Ca-betahydroxy-beta-methylbutyrate (Topic) OR Ca-HMB (Topic) and Preprint Citation Index (Exclude – Database)  #4: randomized controlled trial (Topic) OR randomized (Topic) OR placebo (Topic) OR random (Topic) OR randomised (Topic) and Preprint Citation Index (Exclude – Database)  #5: (#1 AND #3) AND #2 AND #4 and Preprint Citation Index (Exclude – Database) | 37 |
| **Science Direct** | ("Sarcopenias" OR "Sarcopenia") AND ("Exercise" OR "Exercises" OR "physical activity" OR "activities physical" OR "activity physical" OR "physical activities" OR "exercise physical" OR "exercises physical" OR "physical exercise" OR "physical exercises" OR "acute exercise" OR "acute exercises" OR "exercise acute" OR "exercises acute" OR "exercise isometric" OR "exercises isometric" OR "isometric exercises" OR "isometric exercise" OR "exercise aerobic" OR "aerobic exercise" OR "aerobic exercises" OR "exercises aerobic" OR "exercise training" OR "exercise trainings" OR "training exercise" OR "Training" OR "train" OR "trained" OR "Trainings" OR "trains") AND ("beta-hydroxy-beta-methylbutyrate" OR "beta-hydroxy-beta-methylbutyrate" OR "beta-hydroxy-beta-methylbutyrate" OR "3 hydroxyisovaleric acid" OR "HMB" OR "beta-hydroxyisovaleric acid" OR "calcium beta hydroxy beta methylbutyrate" OR "Ca-HMB" OR "beta-hydroxyisovaleric acid") AND ("randomized controlled trial" OR "randomized" OR "placebo") | 63 |

* All searches were carried out on April 1, 2024.

# Table S3. Example Excluded studies

|  | **Reference** | **Reason** |
| --- | --- | --- |
| 1 | Bauer JM, Verlaan S, Bautmans I, Brandt K, Donini LM, Maggio M, McMurdo ME, Mets T, Seal C, Wijers SL, Ceda GP, De Vito G, Donders G, Drey M, Greig C, Holmbäck U, Narici M, McPhee J, Poggiogalle E, Power D, Scafoglieri A, Schultz R, Sieber CC, Cederholm T. Effects of a vitamin D and leucine-enriched whey protein nutritional supplement on measures of sarcopenia in older adults, the PROVIDE study: a randomized, double-blind, placebo-controlled trial. J Am Med Dir Assoc. 2015 Sep 1;16(9):740-7. doi: 10.1016/j.jamda.2015.05.021 | Supplement not based on HMB |
| 2 | Malafarina V, Uriz-Otano F, Malafarina C, et al. Effectiveness of nutritional supplementation on sarcopenia and recovery in hip fracture patients. A multi-centre randomized trial[J]. Maturitas, 2017, 101 42-50. | No exercise intervention |
| 3 | Malafarina V, Uriz-Otano F, Gil-Guerrero L, et al. Study protocol: High-protein nutritional intervention based on β-hydroxy-β-methylbutirate, vitamin d3 and calcium on obese and lean aged patients with hip fractures and sarcopenia. The hiperprot-ger study[J]. Maturitas, 2013, 76 (2): 123-128. | Study protocol |
| 4 | Ma SL, Wu J, Zhu L, Chan RS, Wang X, Huang D, Tang NL, Woo J. Peripheral Blood T Cell Gene Expression Responses to Exercise and HMB in Sarcopenia. Nutrients. 2021 Jul 5;13(7):2313. doi: 10.3390/nu13072313. | Different outcome measures |
| 5 | Din USU, Brook MS, Selby A, et al. A double-blind placebo controlled trial into the impacts of hmb supplementation and exercise on free-living muscle protein synthesis, muscle mass and function, in older adults[J]. Clinical nutrition (Edinburgh, Scotland), 2019, 38 (5): 2071‐2078. | Subjects without sarcopenia |
| 6 | Wu TT, Chen QL, Lin XX, et al. Effects of a multilevel intervention of resistance training with or without beta-hydroxy-beta-methylbutyrate in medical ICU patients during entire hospitalisation: a four-arm multicentre randomised controlled trial. Crit Care. 2023 Dec 15;27(1):493. doi: 10.1186/s13054-023-04698-x. | Subjects were partially sarcopenia |
| 7 | Liao C-D, Tsauo J-Y, Wu Y-T, et al. Effects of protein supplementation combined with resistance exercise on body composition and physical function in older adults: A systematic review and meta-analysis[J]. The American Journal of Clinical Nutrition, 2017, 106 (4): 1078-1091. | Meta-analysis |
| 8 | Wang J, Cui C, Chim YN, et al. Vibration and β-hydroxy-β-methylbutyrate treatment suppresses intramuscular fat infiltration and adipogenic differentiation in sarcopenic mice[J]. Journal of Cachexia Sarcopenia and Muscle, 2020, 11 (2): 564-577. | Animal experiment |
| 9 | Avola M, Mangano GRA, Testa G, et al. Rehabilitation strategies for patients with femoral neck fractures in sarcopenia: A narrative review[J]. Journal of Clinical Medicine, 2020, 9 (10): 1-16. | Narrative review |
| 10 | Pereira S L, Davis G J , Luo M ,et al. LB010-SUN hydro-β-methylbutyrate (HMB) modulates circulating biomarkers in older adults on extended bedrest (BR) [C]. Clinical Nutrition, 2013, 32(Suppl 1):S226.DOI:10.1016/S0261-5614(13)60588-9 | Meeting abstract |
| 11 | Cádiz Gurrea MdlL, Soares S, Jiménez FJL, et al. Chapter 4 - effects of nutritional supplements on human health. Nutraceuticals and natural product pharmaceuticals, 2019, 105-140, https://www.sciencedirect.com/science/article/pii/B9780128164501000040. | Book |
